# Rapid prototyping mixed-signal development kit for tactile neural computing

**DOI:** 10.3389/fnins.2023.1118615

**Published:** 2023-02-07

**Authors:** Vasudev S. Mallan, Anitha Gopi, Chithra Reghuvaran, Aswani A. Radhakrishnan, Alex James

**Affiliations:** ^1^School of Electronics Systems and Automation, Digital University Kerala, Thiruvananthapuram, Kerala, India; ^2^Indian Institute of Information Technology and Management, Kerala, India

**Keywords:** computing arrays, field programmable analog arrays, leaky integrate and fire neuron, tactile sensing system, field programmable gate arrays

## Abstract

Intelligent sensor systems are essential for building modern Internet of Things applications. Embedding intelligence within or near sensors provides a strong case for analog neural computing. However, rapid prototyping of analog or mixed signal spiking neural computing is a non-trivial and time-consuming task. We introduce mixed-mode neural computing arrays for near-sensor-intelligent computing implemented with Field-Programmable Analog Arrays (FPAA) and Field-Programmable Gate Arrays (FPGA). The combinations of FPAA and FPGA pipelines ensure rapid prototyping and design optimization before finalizing the on-chip implementations. The proposed approach architecture ensures a scalable neural network testing framework along with sensor integration. The experimental set up of the proposed tactile sensing system in demonstrated. The initial simulations are carried out in SPICE, and the real-time implementation is validated on FPAA and FPGA hardware.

## 1. Introduction

Field-Programmable Analog Arrays (FPAA) are the analog counterparts to the more popular Field-programmable gate arrays (FPGA) (Farsa et al., 2019; Azghadi et al., 2020; Yu et al., 2020). The ability to program and configure FPGAs has resulted in numerous applications being developed in a short period. In this modern era, reconfigurable computing largely considers only the digital VLSI implementations and the fact is that mostly people turn a blind eye toward the possibilities with analog computing (Azghadi et al., 2020; Yu et al., 2020; García Moreno et al., 2021). In contrast, most sensors detect signals in the analog domain and require analog interface circuits for further processing. Furthermore, the progress in edge artificial intelligent computing has forced the inclusion of more computing modules next to sensors for efficient data processing. This makes a strong case for considering analog computing as a natural approach to be used next to sensors.

The FPAA processors consist of a set of reconfigurable analog circuit blocks (Sekerli and Butera, 2004). These blocks consist of switched capacitor logic that can be programmed to realize various analog computing operations. Such a system can easily build multipliers, adders, and integral and differential operations. FPAA applications that involve signal processing or data converters can find immediate applications to be used in conjunction with sensors. Another possibility is to implement intelligent data processing using analog neural networks.

Various neural networks and neuron models are implemented with FPAA (Rocke et al., 2005; Maher et al., 2006; Schlottmann and Hasler, 2011). Commercially available FPAA AN221E04 was used to build a 2-input, 1-output, 5-intermediate neuron model in Rocke et al. (2005) and Maher et al. (2006). A feed-forward neural network trained with the MNIST dataset is implemented using the AN231E04 FPAA Anadigm in García Moreno et al. (2021). The implementation of FPAA of neuron models such as Hodgkin Huxley and FitzHugh-Nagumo neurons was successfully tested in the past (Zhao and Kim, 2007; Joubert et al., 2012; Khanday et al., 2019; Natarajan and Hasler, 2019). These success stories indicate the wide possibilities with FPAA-based computing.

This paper explores the combined use of FPGA and FPAA arrays as a prototyping tool to test an integrated solution for real-time tactile sensing, recognition, and classification. This uses the popular neuron model, “Leaky Integrate and Fire,” for the first neural network layer implemented on FPAAs. The remaining neural network layers are implemented in the digital domain using FPGAs. This takes the best of both worlds, where the sensing layer is analog while the remaining layers responsible for classification are implemented in FPGAs. The major contributions of the work are (1) to convey the practical demonstration of the use of tactile sensing with FPAAs, (2) to show a unique scalable array architecture built with FPAAs for near-sensor computing, and (3) to exhibit the possibilities of mixed-signal pipelines sequentially built on FPAA and FPGA to create large-scale neural networks next to sensors.

## 2. Tactile sensing with mixed-mode neural computing

**Figure 1:**
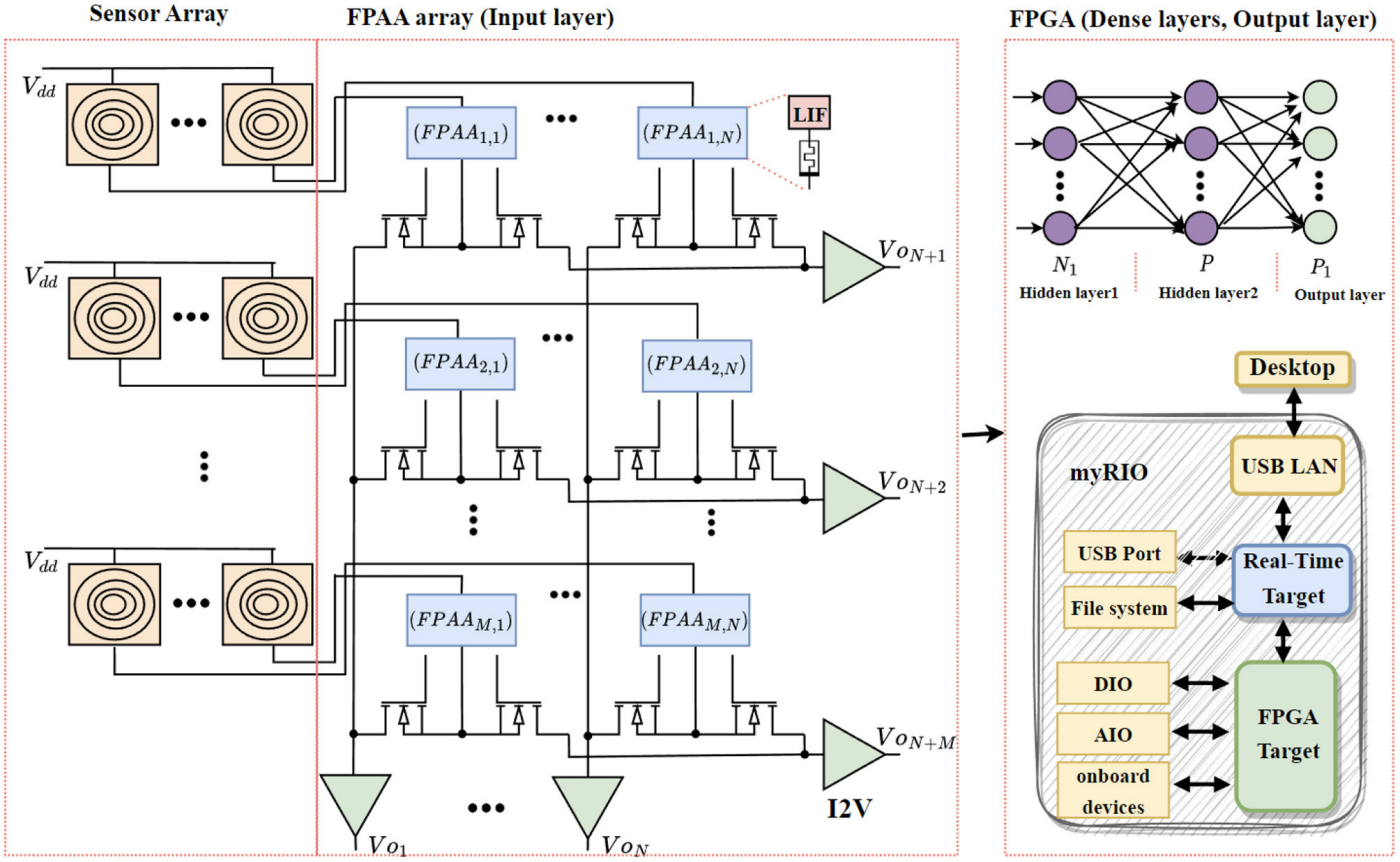
Block diagram representation of the proposed mixed signal developmental kit for tactile sensing system, where DIO and AIO denote digital input/output and analog input/output respectively of FPGA. *N*_1_ = *N*+*M*.

**Figure 2:**
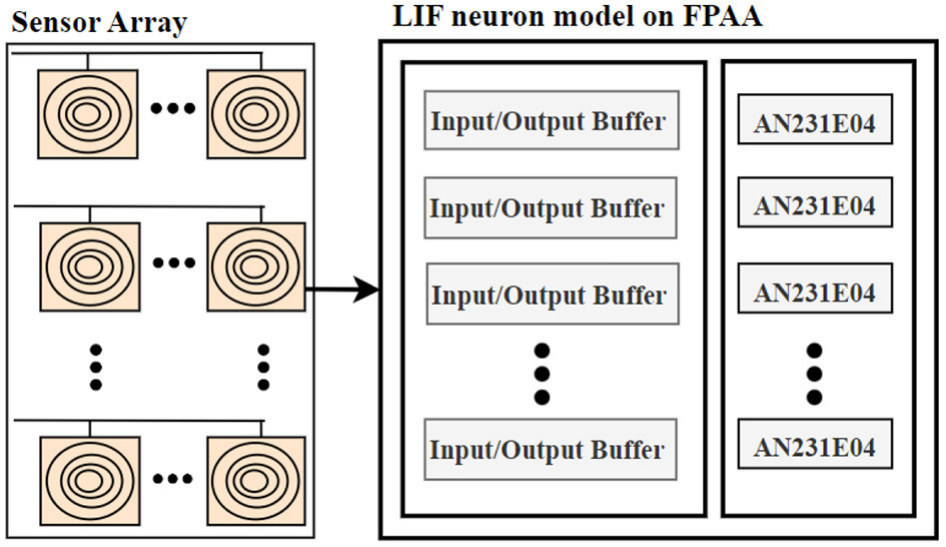
Block diagram representation sensor neuron subsystem, sensor array and LIF neuron model implemented on FPAA.

Figure 1 shows the overall block diagram of the proposed system. The input layer of the neural network representing the sensory neurons is implemented with a touch sensor and FPAA, while the rest of the dense neural layers are implemented with an FPGA module. The block diagram representation of the proposed sensor-neuron submodule is shown in Figure 2.

The neural network's input layer consists of the sensor-neuron module in a weighted crossbar array configuration. The weighted summation is done based on the combination of a transistor as a switch, a memristor for non-volatile programmable memory, and a touch sensor-neuron module, forming a node in a crossbar array configuration. The first layer (Figure 1) consists of the nodes with sensor neurons, memristors, and transistors that can perform dot-product computations on the crossbar. The sensor-neuron output is weighted and summed, and the output is read across the horizontal and vertical lines of the crossbars.

Neural networks extensively use weighted summation operations, where the weights are optimized against an objective error function. Minimizing errors is essential to maximize recognition accuracy. This optimization is referred to as neural network training, and the most popular approach is to use gradient descent implemented through the backpropagation algorithm. Training is performed on the cloud server using custom-made Python scripts that use data from crossbar arrays. The trained parameters of the neural network are extracted, and subsequent dense layers are implemented in the FPGA.

### 2.1. Crossbar array

The Leaky Integrate and Fire Neuron Model (neuron model) is a well-known neuron model to emulate the action potentials in a neuron (Nahmias et al., 2013; Dutta et al., 2017). The neuron model represents a neuron as a parallel combination of a leaky resistor, a capacitor, and a current source. A current source *I*(*t*) in the neuron model is used as the input of synaptic current to charge the capacitor to produce a potential *V*(*t*). The current equations in the neuron model are given by Dutta et al. (2017).


(1) $$\begin{array}{l} I\left(t\right)=C\frac{\text{d}V\left(t\right)}{\text{d}t}+\frac{\left(V\left(t\right)-V_{rest}\right)}{R} \end{array}$$

Where *C* is the membrane capacitance, *V*(*t*) represents the membrane potential at time *t*, *R* is the membrane resistance and *I*(*t*) is the input current. If the membrane potential exceeds *V*_*ref*_, then a spike is generated after releasing the spike, and *V*(*t*) is reset to a resting potential *V*_*rest*_ = *V*_*t*_ − *V*_*ref*_. These equivalent circuits are implemented using Configurable Analog Modules (CAMs) in FPAA. The desired results were obtained by setting the chip clock frequency to 4 MHz and the CAM frequency to 62.5 kHz.

**Figure 3:**
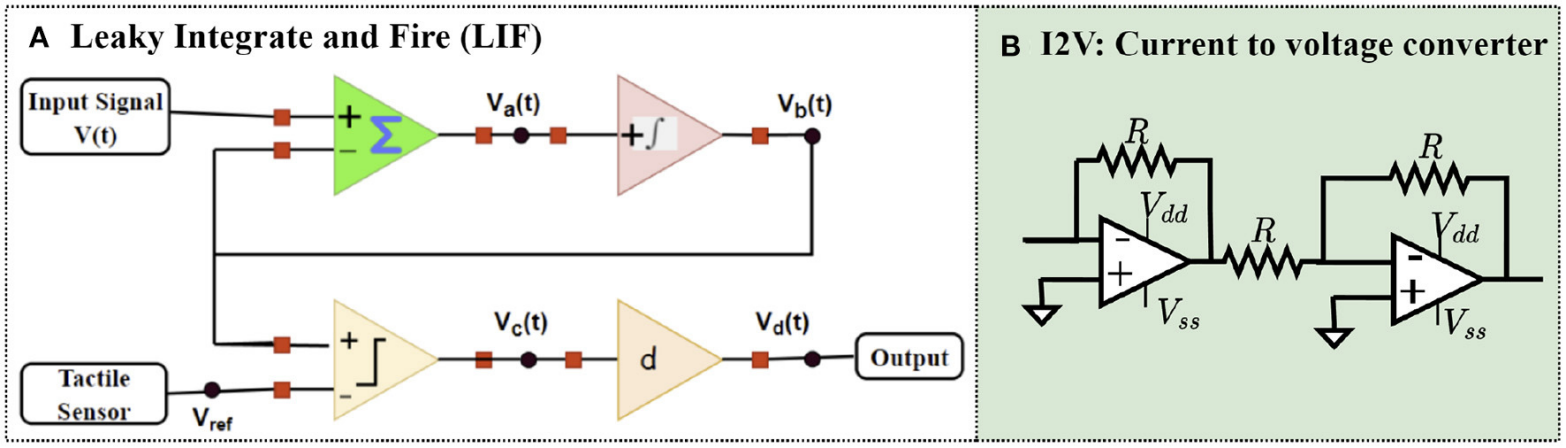
**(A)** FPAA equivalent circuit model of LIF system and **(B)** current to voltage converter (I2 V).

Figure 3 shows the equivalent FPAA blocks proposed for the neuron model. The proposed neuron model in FPAA consists of four CAMs, sum/difference, integrator, comparator, and differentiator. Sum/difference collects the sum of input signals, *V*_*a*_(*t*). Here, the sensor voltage will act as a reference voltage changing the threshold voltage from 0 to *V*_*ref*_. *V*_*a*_(*t*) is passed through a low-pass filter, and these signals are integrated over time. This CAM functionality is similar to that of the Soma part of the neuron. The integrated signals *V*_*b*_(*t*) are fed to a comparator that compares with a threshold voltage, *V*_*ref*_(*t*), from the tactile sensor. *V*_*ref*_(*t*) is obtained as a result of the sensation of touch in the tactile sensor. This signal is considered the reference signal. The output of the comparator can be mathematically represented as:


(2) $$\begin{array}{l} V_{c}\left(t\right)=\left\{\begin{array}{cc} V_{b}\left(t\right), & V_{b}\left(t\right)>V_{ref}\\ -V_{b}\left(t\right), & V_{b}\left(t\right)<V_{ref} \end{array}\right\} \end{array}$$

The signal *V*_*c*_(*t*) is differentiated to generate the spikes that trigger the sensation of touch similar to the human body. In summary, the spikes were generated by human contact with the touchpad of the sensor. The signal from the sensor is directly fed to the neuron model as the reference voltage (*V*_*ref*_).

Figure 1 shows the implementation of crossbar arrays that incorporate a sensor-neuron submodule. Each cell of the proposed sensor-neuron crossbar array consists of a sensor-neuron module, a memristor for weighted multiplication of sensor-neuron output, and 2 transistors. The two transistors avoid sneak path currents in the horizontal and vertical lines and enable readouts in the vertical and horizontal lines. The read-out currents can be calculated as follows:


(3) $$\begin{array}{l} i_{x}\left(t\right)=\underset{j,k}{\sum }\left\{a_{j,k}+b_{j,k}\right\}g_{j,k}V_{c}\left(t\right) \end{array}$$

**Figure 4:**
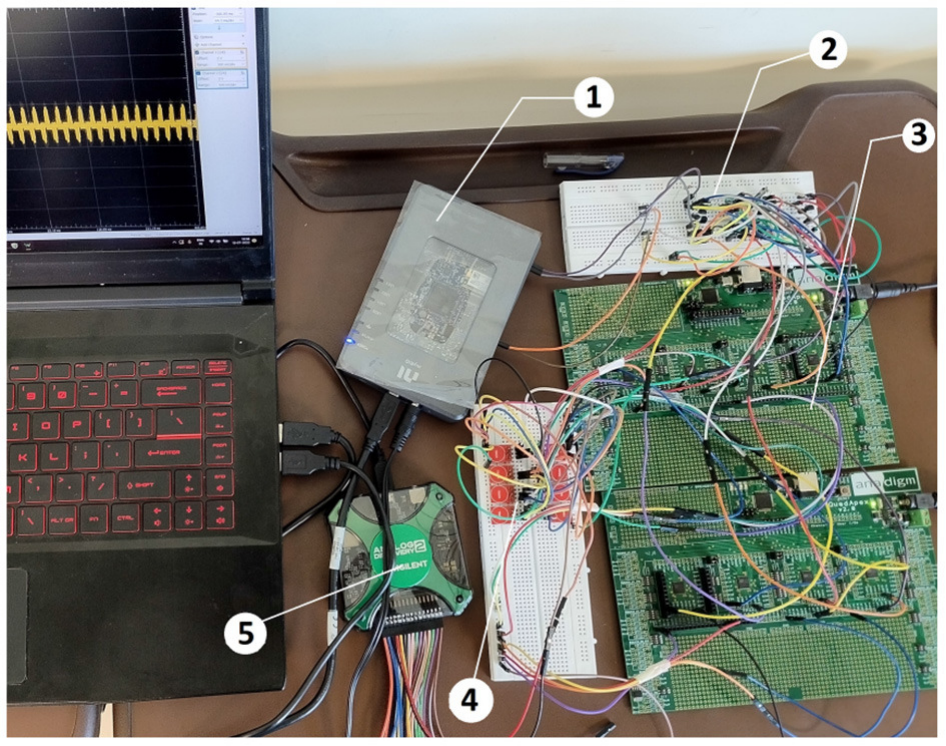
Experimental setup of sensor-LIF tactile sensing system (1) NI myRIO 1900, (3) Anadigm FPAA board with AN231E04 Chip, (2), (3), (4) Sensor-LIF crossbar array, and (5) USB Oscilloscope.

Where *j* and *k* denote the row and column number, respectively. *a*_*j,k*_, *b*_*j,k*_ ∈ {1, 0} represents the switching of the transistor. For horizontal readouts, {*a*_*j,k*_ = 1, *b*_*j,k*_ = 0} and for vertical readouts {*a*_*j,k*_ = 0, *b*_*j,k*_ = 1}. The weighted summation of currents *i*_1_, *i*_2_, ...*i*_*M*+*N*_ give unique representation of each tactile sensation. Readout currents are converted to voltages using current-to-voltage converters, as shown in Figure 3. The memristor in the sensor-neuron crossbar arrays can be reprogrammed based on tactile applications. This experiment employs a fully integrated memristive model in Jin and Cui (2019). The FPAA equivalent circuit implementation is shown in Figure 4A, which emulates the characteristics of a fully integrated memristive model. Figure 4B shows the pinched hysteresis curve of the memristive model with device parameters *R*_*ON*_ = 1*KΩ* and *R*_*OFF*_ = 100*KΩ*. The output of the LIF circuit is given as the input to this memristive model, and the output of these memristors is read out using the FPAA crossbar. As shown in Figure 1, the horizontal and vertical read-outs of the crossbar form the input to the next dense layer.

### 2.2. FPGA implementation of dense layers

For detection, the sensed data is used to train dense layers using Python scripts. The measured current values from sensor-neuron crossbar arrays are prone to different types of noise, and a small variation in any of the current values can lead to incorrect classification. In order to avoid the noise effect on the current values, the measured values are prepossessed to a normalized value using the mean filter. It removes unwanted boundary values and takes the average among the whole sample. These values are used for training the dense layers using Python scripts and programmed on-to FPGA.

The neural computations of each dense layer are calculated using the formulas *Y* = *XW*+*B*, where *X* and *W* are the input and the weight values of sizes *M*_1_ × *N*_1_ and *N*_1_ × *P*, respectively. *B* is the bias, and *Y* is the output, both of which have a size of *M*_1_ × *P*. Here, *X* is the output of sensor-neuron crossbar array, i.e., *X* = {*Vo*_1_, *Vo*_2_...*Vo*_*M*_1_*N*_1__}. The trained weights, *W*, and the bias, *B*, are stored in memory. The *X* and *W* are converted from the matrix to arrays of sizes 1 × (*M*_1_*N*_1_) and 1 × (*N*_1_*P*) using the reshape array block. The single layer of ANN can be implemented in myRIO FPGA using LABVIEW using the steps listed in Table 1. The LABVIEW implementation consists of real-time FPGA target applications. The real-time target application provides an interactive environment to start running FPGA VI and with the desktop computer. The Analog Input/Output (AIO) data read, memory read, FIFO definition, start, and close of FPGA VI target are done in the real-time target application. Whereas FPGA target application synthesizes the circuit on FPGA and generates the bitstream file. The dense layer computations are done in FPGA VI.

**Table 1:** Steps for implementing a single dense layer in myRIO FPGA using LABVIEW.

	**Real-time target application**
1	Open While loop
2	Read *W* and *B* from the memory
3	Capture the data from AIOs and write to an array *X*
4	Reshape *X*, *W*, and *B* from matrix to arrays
5	Define 3 FIFOs of type Host to Target DMA for *X*, *W*, and *B*
6	Define 1 FIFO of type Target to Host DMA for *Y*
7	Open FPGA VI reference
8	Write *X*, *W*, and *B* arrays to the DMA FIFOs from the host VI
	using FIFO.Write (Invoke Method)
9	Read *Y* of the DMA FIFO using FIFO.Read (Invoke Method)
10	Reshape *Y* to matrix and display
11	Close FPGA VI reference
12	Close While loop
	**FPGA target application**
13	Open Single cycle timed loop
14	Read *X*, *W*, and *B* using Read (FIFO method)
15	Perform *U* = *X* × *W* using matrix multiply function
16	Build matrix *B* with the same size as *U*
17	Perform *Y* = *U*+*B* using high throughput ADD function
18	Split **Y** and write to FIFO using write (FIFO method)
19	Close Single cycle timed loop

## 3. Results and discussions

**Figure 5:**
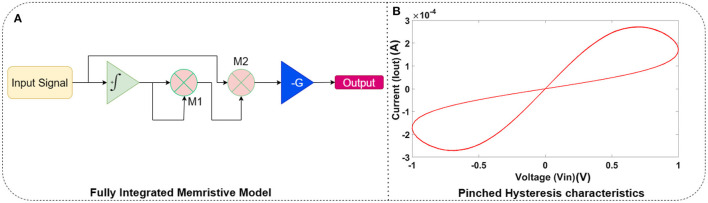
**(A)** FPAA equivalent circuit model of fully integrated memristive model (Jin and Cui, 2019) and **(B)** The pinched hysteresis characteristics of the memristive model.

The proposed mixed-mode neural computing is experimentally demonstrated on a system for identifying braille and morse code symbols (Figure 5). The sensor-neuron crossbar array's input layer acts as a tactile patch for blind users to press the braille and the morse code characters. In the Braille system, each character is represented by 6 points (D1, D2, D3, D4, D5, D6) (Chithra et al., 2022). Some of the braille symbols have the same representations. For example, the character *A* and the number 1 have the same representations. Hence, we use two additional dots (D7, D8) to differentiate them. Thus, we represent 125 braille characters. In the case of Morse code, the repetitive combination of dots and dashes forms alphabets and numbers. Here, 10 dots are used for representing morse code each column representing either dots or dashes. The selection dots (D11, D12) represent braille and morse code selection. The designed system implemented 62 characters (capital letters, small letters, and numbers) for morse code. Hence the data set consists of 40 instances of 187 different symbols of braille and morse code. Hence, the tactile sensing system is implemented using a sensor-neuron crossbar size of 6 × 2. Each dot represents one cell in the sensor-neuron crossbar array. Table 2 shows the characters implemented in the proposed tactile sensing system. The difference in character implementations of braille and morse code are presented in Table 3.

**Table 2:** Braille and morse code character implementation on LIF-neuron crossbar array, 1/0 denotes touch sensor is pressed/not pressed.

**{*D*_11_, *D*_12_}**	**{*D*_7_, *D*_8_}**	**Characters**
0, 0	0, 0	Braille alphabet capital (27 symbols)
0, 0	0, 1	Braille small (26 symbols)
0, 0	1, 0	Braille words (46 words)
0, 0	1, 1	Braille numbers, punctuation & symbols (26
		symbols)
1, 1	Morse alphabet capital (26 symbols)	
0, 1	Morse Small (26 symbols)	
1, 1	Morse code numbers (10 symbols)	

**Table 3:** Braille and morse character implementation using the sensor array patch, 1/0 denotes touch sensor is pressed/not pressed.

**Character**	**{*D*_1_, *D*_2_, *D*_3_, *D*_4_, *D*_5_, *D*_6_, *D*_7_, *D*_8_, *D*_9_, *D*_10_, *D*_11_, *D*_12_}**
**Braille characters**	
A	{1, 0, 0, 0, 0, 0, 0, 0, 0, 0, 0, 0}
a	{1, 0, 0, 0, 0, 0, 1, 0, 0, 0, 0, 0}
1	{1, 0, 0, 0, 0, 0, 1, 1, 0, 0, 0, 0}
**Morse code**	
A	{1, 0, 0, 1, 0, 0, 0, 0, 0, 0, 1, 1}
a	{1, 0, 0, 1, 0, 0, 0, 0, 0, 0, 1, 0}
1	{1, 0, 0, 1, 0, 1, 0, 1, 0, 1, 1, 1}

### 3.1. Training—Simulations

The preliminary neuron circuit is simulated using SPICE tools and the equivalent circuit of the same is implemented in FPAA using different CAMs. The real-time implementation of neuron sensor crossbar arrays for the Braille character recognition system uses a cluster of 12 FPAA chips. The weighted summation of the current from each cell is taken through the HL and VL lines. The input layer consists of 6 preneurons and 8 postneurons. The programming of weights requires training the neurons taking into account hardware variability. Simulations are carried out using equivalent models to estimate the weight values. This experiment uses a capacitive touch sensor module (TTP223 touch-sensing IC) and fully integrated memristive model. The generated dataset is used to train the subsequent dense layers using Python program.

**Figure 6:**
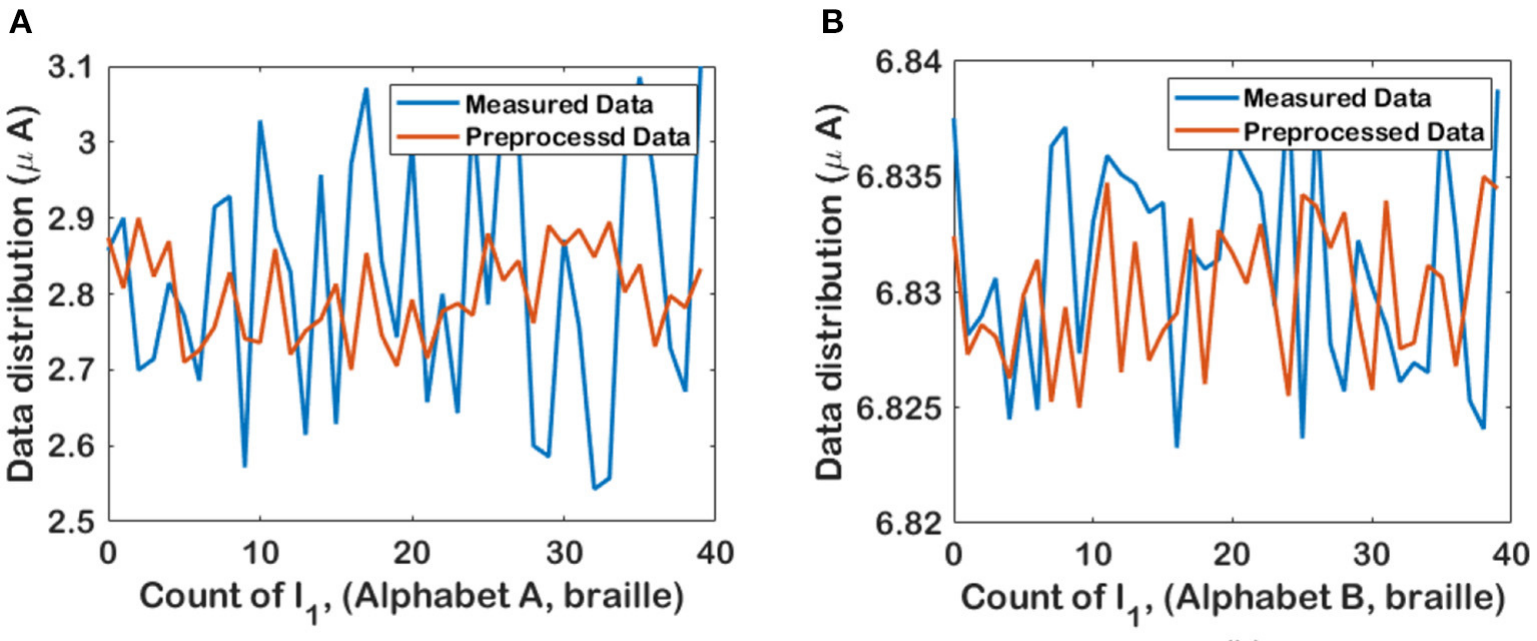
Min-max normalized data for *i*_1_ readout of LIF-TMS crossbar array for **(A)** Character A and **(B)** Character B.

The measured values of FPAA crossbar output are prone to different types of noise. The noise in the measured data affects the boundary points of 187 class of symbols of braille and morse code. Any small deviations will lead to change in the whole feature set combination. Hence a min-max scalar based preprocessing technique is adopted to remove the noise factors that might be affected at the boundary points. In min-max normalization, the noisy data is scaled up/down using a range based on averaging. Figure 6 shows the first column current values for characters A and B, respectively. For each character, we take 40 samples. The measured data is normalized into a symmetric range after preprocessing. For example, the data range of the measured value of character A is between 2.5 and 3.1, as shown in Figure 6. With min-max preprocessing, the data range for character A is limited between 2.7 and 2.9. This helps to remove the noise factors affecting the boundary points.

The training for the FPAA crossbar data is done with and without preprocessing technique. The dataset consists of 7480 samples of braille and morse code, i.e., 40 samples of 187 symbols. The generated data set is used to train the subsequent dense layers using the Python program. The trained model consists of 2 hidden layers and an output layer. The first layer has 8 × 14 nodes with relu activation function. The output layer uses a softmax activation function of 187 node size. The trained parameters of the dense layer are then implemented on myRIO FPGA for the real-time implementation.

### 3.2. Inference—Hardware implementation

**Figure 7:**
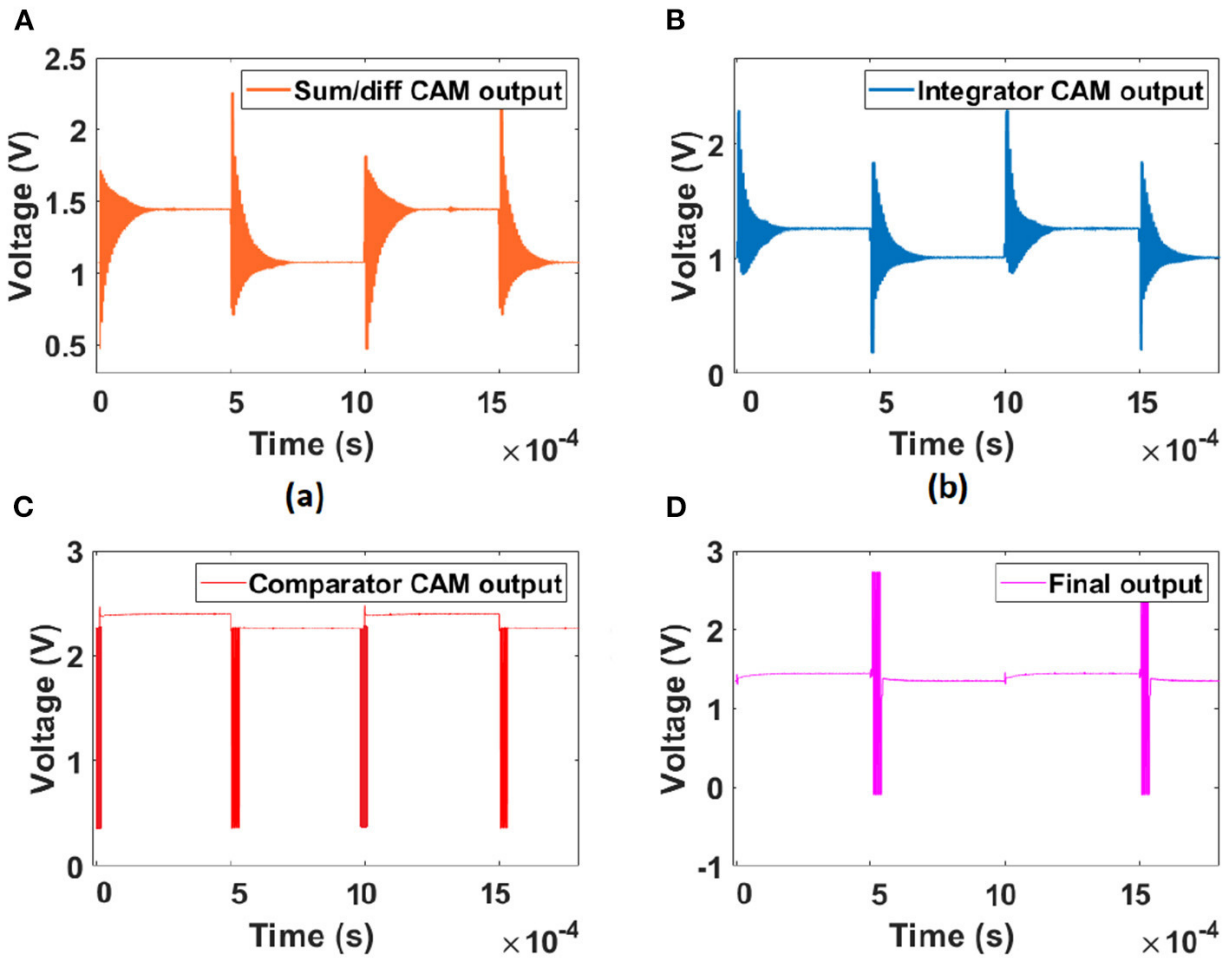
Hardware implementation output of LIF FPAA implementation with an offset of 1 V: **(A)** Sum/Diff CAM output, **(B)** integrator CAM output, **(C)** the Comparator CAM output with a reference voltage of 0.3 V, and **(D)** the final output of the sensor-LIF module.

The proposed design is implemented on the commercially available Anadigm AN231E04 IC. The parasitic capacitance of the hardware components introduces noise in the output of each of the CAM modules, as shown in Figure 7. Hence, the output spikes have noise content in them as shown in Figure 7D. The chip clock frequency is 4 MHz, and each CAM clock frequency is 62.5 KHz. The spikes occur only when someone touches the touchpad of the tactile/touch sensor. This reference potential acts as a trigger for the sensor neuron model to generate a spike.

The layer1 output is read through AIO pins on the myRIO. The output of the sensor-neuron crossbar array is measured and used to train the dense layers to perform the classification. The noise in the measured data from the sensor-neuron crossbar array affects the boundary points of 187 classes of symbols of braille and morse code. Any small deviations will lead to a change in the whole feature set combination. Hence a min-max scalar-based preprocessing technique is adopted to normalize the measured values (more details in the Supplementary material). Training of the sensor data is done with and without noise removal. The trained ANN model contains five dense layers with input, hidden, and output layers. The relu activation function is used for all layers, and the output layer uses the softmax activation function (Krestinskaya et al., 2020; Newns et al., 2020).

**Figure 8:**
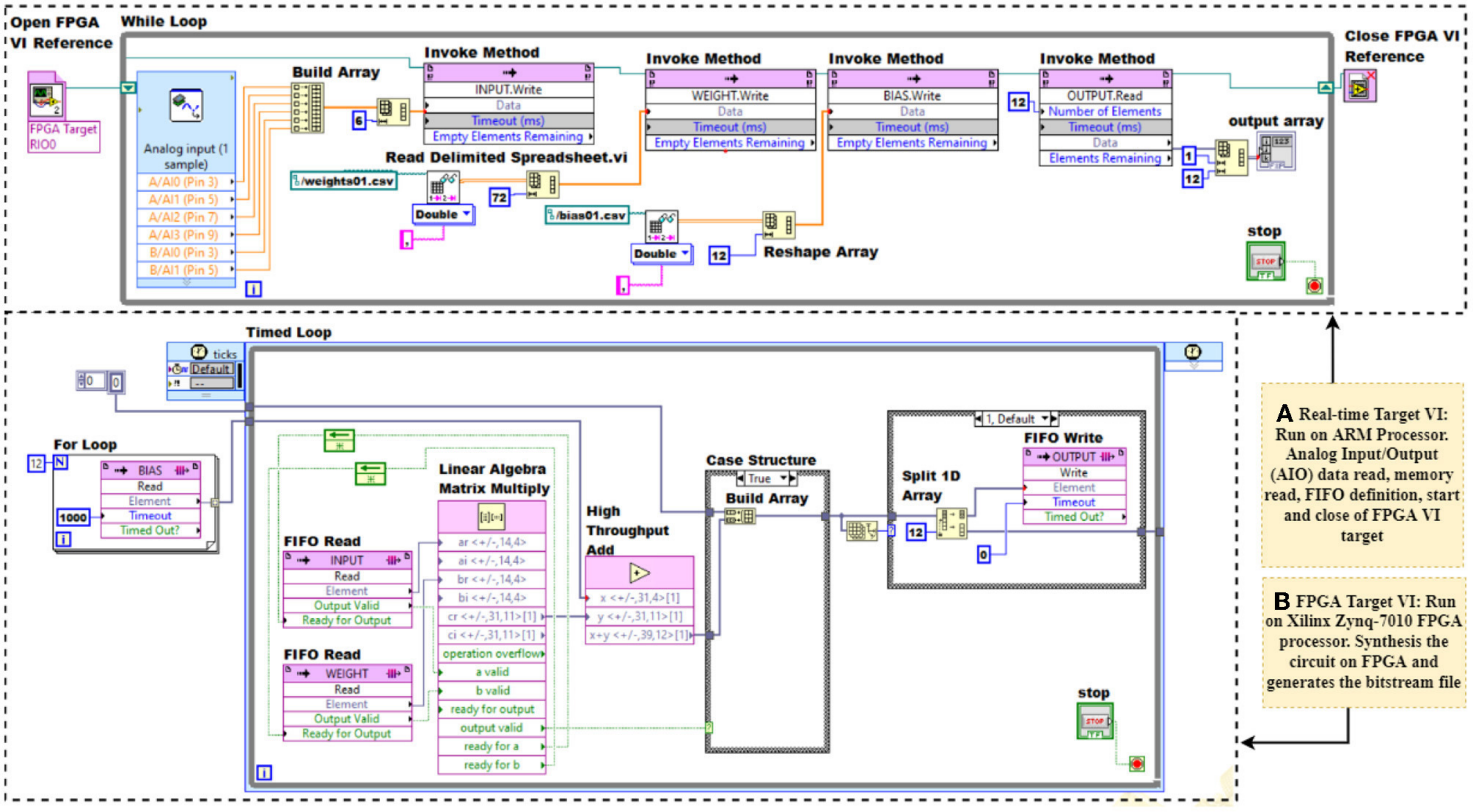
Single dense layer implementation on LABVIEW application. **(A)** Real-Time target VI and **(B)** FPGA target VI.

The trained model is then implemented on myRIO FPGA for real-time implementation. As discussed in Table 1, the implementation consists of real-time target VI and FPGA target VI. The LABVIEW application of real-time target VI and FPGA target VI is shown in Figure 8. The open FPGA VI reference will cause FPGA VI to start running (Table 1). The while loop makes the system run continuously for real-time applications. The single-cycle timed loop structures are always used in an FPGA VI, which will execute all functions within one tick of the clock, here we use a 40 MHz global clock. The linear algebra matrix multiply function block and high-throughput add function block work only inside the single-cycle timed loop. For dense layer1, *M*_1_ = 1, *N*_1_ = 8 and *P* = 14. Correspondingly, the FIFO depths are 8, 112 and 14, respectively, for *X*, *W*, and *B*. The output of the linear algebra matrix multiply function block is a column vector. Hence, the bias values read from the FIFO are converted to arrays of size *M*_1_ × *P*, here 1 × 8. The bias addition is done using the high-throughput add function block. The output of the present layer forms the input to the subsequent layers. The graphical programming of real-time target VI and FPGA VI is presented in Figure 8.

Table 4 shows the average relative error, *RE*_*avg*_ of hardware dense layer output in comparison with software results. *RE*_*avg*_ can be calculated as $\frac{1}{P_{1}}\overset{P_{1}}{\underset{l=1}{\sum }}\frac{\left|Ys_{l}-Yh_{l}\right|}{Ys_{l}}$, where *Ys*_*l*_ and *Yh*_*l*_ denote the software and hardware results of the output layer. The table shows the relative output error for varying FIFO integer length on myRIO. Each symbol in the braille alphabet has different combinations of dots, i.e, for braille character “A” all the FPAA array output is zero except *Vo*_1_ and *Vo*_3_. This creates sparsity in the subsequent dense layers and output layer. Whereas for braille character “Y,” only *Vo*_7_ and *Vo*_8_ are zeros (according to Table 2). Hence there are variations in *RE*_*avg*_ for different symbols as shown in Table 4. The results show that 16 bits representation shows comparable performance with software results. Hence the FIFOs for FPGA implementation are defined for a word length of 16 bits. The system testing accuracy is 65% for braille characters and 75% for morse characters with hardware noise (Table 5). With the preprocessed input data using Min-Max normalization, the performance accuracy is improved to 96 and 98%, respectively, for braille and morse code character detection. Table 5 shows the values of precision (P), recall (R), and F1 score (F1) for 5 characters.

**Table 4:** Average relative error, *RE*_*avg*_, of hardware dense layer output in comparison with software results with varying FIFO integer length.

	**Integer word length**				
**Symbol**	**2 bits**	**4 bits**	**8 bits**	**12 bits**	**16 bits**
A	0.13	0.03	0.01	0	0
D	0.46	0.34	0.23	0.13	0.03
I	0.43	0.22	0.11	0.06	0.01
T	0.89	0.56	0.34	0.19	0.06
Y	1.05	0.72	0.46	0.26	0.09

**Table 5:** Testing accuracy of Braille and morse recognition system with sensor-neuron crossbar array.

	**Braille characters**						**Morse code**					
	**With noise** **Acc** = **65%**			**With filter** **Acc** = **96%**			**With noise** **Acc** = **75%**			**With filter** **Acc** = **98%**		
	**P**	**R**	**F1**	**P**	**R**	**F1**	**P**	**R**	**F1**	**P**	**R**	**F1**
A	1.0	1.0	1.0	1.0	1.0	1.0	0.12	1.00	0.22	0.60	1.00	0.57
D	0.33	1.0	0.50	0.5	1.0	0.67	0.38	1.00	0.55	1.00	1.00	1.00
J	0.0	0.0	0.0	0.22	1.00	0.36	0.40	1.00	0.50	0.8	1.00	0.89
T	0.56	1.0	0.71	1.0	1.0	1.0	0.6	0.58	0.88	1.00	1.00	1.00
Y	1.0	1.0	1.0	1.0	1.0	1.0	1.00	1.00	1.00	1.00	1.00	1.00
Avg	0.58	0.69	0.60	0.90	0.922	0.88	0.71	0.72	0.72	0.92	0.96	0.95

P, R, F1, and Acc denote precision, recall, F1 score, and accuracy, respectively.

## 4. Discussions

### 4.1. Scalability in rapid prototyping

The proposed mixed-signal development kit is a rapid prototyping solution for different neural computing applications. The paper presents the implementation of braille character and moorse code recognizing system using the same prototype designed. The system can be easily scaled up by adding FPAA arrays and reprogramming the FPGA. This makes the system more flexible in implementing other neural tactile sensing applications.

### 4.2. Neural architecture optimization

The proposed mixed signal processing helps to reduce the required power consumption by optimizing the neural architecture. In the conventional method, the sensed data from each sensors are transmitted for neural computing. i.e., for an *M* rows and *N* columns sensor array, the conventional method takes *M* × *N* inputs for neural processing. Whereas, our proposed method shown in Figure 1 only needs *M*+*N* inputs for neural processing. The reduction in number of inputs directly reduces the size of neural network architecture. Table 6 demonstrates the neural architectural comparison of existing mixed signal prototyping with the conventional implementation techniques using either FPAA or FPGA. The results in the table show there is approximately 30% reduction in the neural architecture size for the mixed signal implementation to achieve the same accuracy of 96% for the braille and 98% for the morse code character recognition system.

**Table 6:** Mixed signal development kit for tactile application: a comparison with the existing implementation methods.

	**Number of neurons**		
**Method**	**Hidden layer1**, *N*_1_	**Hidden layer2**, *P*	**Output layer**, *P*_1_
FPAA (García Moreno et al., 2021)	12	19	187
FPGA (Azghadi et al., 2020)	12	19	187
Proposed (FPAA+FPGA)	8	14	187

## 5. Conclusion

The mixed signal hardware for a neural network based on sensor-neuron crossbars using an FPAA and FPGA cluster is the focus of the study in the article. The sensor-neuron crossbar neural network shows the analog domain computation for the input layer and the digital domain computation for dense layers. An equivalent LIF circuit is designed using CAM and is implemented on Anadigm AN231E04 ICs. The proposed sensing module is then used to implement a tactile sensing application for a Braille and Morse character identification system. The simulation results show that the proposed model is accurate and power-efficient in the temporal domain. The FPAA platform enables complex circuit design much more easily using configurable analog modules. The proposed prototyping approach helps to optimize the mixed-signal sensor-neural network designs before being deployed for on-chip implementations.

## 6. Experimental methods

This experiment uses the TTP223 capacitive touch sensor module. Each sensor in the system is connected to the LIF neuron model implemented on the AN231E04 chip. The equivalent circuit model of the LIF neuron module is designed on the FPAA module using the Anadigm QuadApex development board. Each board has 4 AN231E04 Dynamically Reconfigurable Analog Signal Processors (dpASP) and operates with a clock frequency of 4 MHz. Each dpASP is programmed using Anadigm Designer 2 EDA software. The dynamically reconfigurable analog signal processor operates from a 3.3 V power supply.

The equivalent circuit of the LIF model is designed using CAM modules in Anadigm Designer 2 EDA software. In AD2 software, analog circuits can be implemented using a library of configurable analog modules (CAMs) by setting the chip clock frequency to 4 MHz and the CAM frequency to 62.5 KHz (Joubert et al., 2011).

The dense neural network is implemented on the NI myRIO FPGA processor Xilinx Zynq-7010. LABVIEW FPGA is the software tool used to graphically implement various digital circuits on the FPGA chip. The trained model is then programmed to the NI myRIO-1900 FPGA target. The FPGA target is programmed using LABVIEW FPGA, a software add-on model to the LABVIEW graphical software development environment. LABVIEW FPGA is used to graphically implement various digital circuits on the FPGA chip. MyRIO hardware consists of an ARM microcontroller (real-time target) and a Xilinx Zynq-7010 (FPGA processor). The myRIO is connected to the host computer via USB or Wireless 802.11b,g,n.

## Data availability statement

The original contributions presented in the study are included in the article/Supplementary material, further inquiries can be directed to the corresponding author.

## Author contributions

VM, AG, CR, AR, and AJ contributed to the overall writing, design, and experimentations. All authors contributed to the article and approved the submitted version.

## References

[B1] Azghadi M. R. Lammie C. Eshraghian J. K. Payvand M. Donati E. Linares-Barranco B.. (2020). Hardware implementation of deep network accelerators towards healthcare and biomedical applications. IEEE Transact. Biomed. Circuits Syst. 14, 1138–1159. 10.1109/TBCAS.2020.3036081 33156792

[B2] Chithra R. Aswani A. R. James A. P. (2022). Tms-crossbars with tactile sensing. IEEE Trans. Circuits Syst. II Express Briefs 69, 1842–1846. 10.1109/TCSII.2021.3128376

[B3] Dutta S. Kumar V. Shukla A. Mohapatra N. R. Ganguly U. (2017). Leaky integrate and fire neuron by charge-discharge dynamics in floating-body mosfet. Sci. Rep. 7, 8257. 10.1038/s41598-017-07418-y 28811481 PMC5557947

[B4] Farsa E. Z. Ahmadi A. Maleki M. A. Gholami M. Rad H. N. (2019). A low-cost high-speed neuromorphic hardware based on spiking neural network. IEEE Trans. Circuits Syst. II: Express Briefs 66, 1582–1586. 10.1109/TCSII.2019.289084636009754

[B5] García Moreno D. Del Barrio A. A. Botella G. Hasler J. (2021). A cluster of fpaas to recognize images using neural networks. IEEE Transact. Circuits Syst. II: Express Briefs 68, 3391–3395. 10.1109/TCSII.2021.3077392

[B6] Jin J. Cui L. (2019). AFully integrated memristor and its application on the scroll-controllable hyperchaotic system. Complexity 2019, 4106398. 10.1155/2019/4106398

[B7] Joubert A. Belhadj B. Héliot R. (2011). “A robust and compact 65 nm lif analog neuron for computational purposes,” in 2011 IEEE 9th International New Circuits and Systems Conference (Bordeaux: IEEE), 9–12. 10.1109/NEWCAS.2011.598120

[B8] Joubert A. Belhadj B. Temam O. Héliot R. (2012). “Hardware spiking neurons design: analog or digital?” in The 2012 International Joint Conference on Neural Networks (IJCNN), 1–5. 10.1109/IJCNN.2012.6252600

[B9] Khanday F. A. Kant N. A. Dar M. R. Zulkifli T. Z. A. Psychalinos C. (2019). Low-voltage low-power integrable cmos circuit implementation of integer- and fractional–order fitzhugh–nagumo neuron model. IEEE Transact. Neural Netw. Learn. Syst. 30, 2108–2122. 10.1109/TNNLS.2018.2877454 30442620

[B10] Krestinskaya O. Choubey B. James A. (2020). Memristive gan in analog. Sci. Rep. 10, 1–14. 10.1038/s41598-020-62676-7 32246103 PMC7125184

[B11] Maher J. Ginley B. M. Rocke P. Morgan F. (2006). “Intrinsic hardware evolution of neural networks in reconfigurable analogue and digital devices,” in 2006 14th Annual IEEE Symposium on Field-Programmable Custom Computing Machines, 321–322. 10.1109/FCCM.2006.53

[B12] Nahmias M. A. Shastri B. J. Tait A. N. Prucnal P. R. (2013). A leaky integrate-and-fire laser neuron for ultrafast cognitive computing. IEEE J. Selected Top. Quantum Electron. 19, 1–12. 10.1109/JSTQE.2013.2257700

[B13] Natarajan A. Hasler J. (2019). “Implementation of synapses with hodgkin huxley neurons on the fpaa,” in 2019 IEEE International Symposium on Circuits and Systems (ISCAS), 1–5. 10.1109/ISCAS.2019.8702489

[B14] Newns D. Solomon P. Cui X. Han J. P. Zhang X. (2020). Analog Circuit for Softmax Function. U.S. Patent 20 200 167 402. Washington, DC: U.S. Patent and Trademark Office.

[B15] Rocke P. Maher J. Morgan F. (2005). “Platform for intrinsic evolution of analogue neural networks,” in 2005 International Conference on Reconfigurable Computing and FPGAs (ReConFig'05), 8–11. 10.1109/RECONFIG.2005.29

[B16] Schlottmann C. R. Hasler P. E. (2011). A highly dense, low power, programmable analog vector-matrix multiplier: the fpaa implementation. IEEE J. Emerg. Selected Top. Circuits Syst. 1, 403–411. 10.1109/JETCAS.2011.2165755

[B17] Sekerli M. Butera R. (2004). “An implementation of a simple neuron model in field programmable analog arrays,” in The 26th Annual International Conference of the IEEE Engineering in Medicine and Biology Society (San Francisco, CA: IEEE) 4564–4567. 10.1109/IEMBS.2004.1404266 17271322

[B18] Yu Y. Wu C. Zhao T. Wang K. He L. (2020). Opu: an fpga-based overlay processor for convolutional neural networks. IEEE Trans. Very Large Scale Integrat. Syst. 28, 35–47. 10.1109/TVLSI.2019.2939726

[B19] Zhao J. Kim Y.-B. (2007). “Circuit implementation of fitzhugh-nagumo neuron model using field programmable analog arrays,” in 2007 50th Midwest Symposium on Circuits and Systems (Montreal, QC), 772–775. 10.1109/MWSCAS.2007.4488691

