# Supplementary material for: Rapid prototyping mixed-signal development kit for tactile neural computing
Source: Front Neurosci. 2023 Feb 7;17:1118615. doi: 10.3389/fnins.2023.1118615 (PMC9941318; doi:10.3389/fnins.2023.1118615)
Supplement: Supplementary file 1 [file Data_Sheet_1.pdf]

Supplementary Material: Rapid Prototyping Mixed-signal Development Kit for Tactile Neural Computing

1 LIF FPAA IMPLEMENTATION: SPECIFICATIONS

In order to achieve temporal sparsity, the sensor output is fed to the LIF block in a LIF-TMS crossbar array (Fig. 1 of the main paper). The LIF neuron model represents a neuron as a parallel combination of a leaky resistor, a capacitor, and a current source Dutta et al. (2017). The equivalent circuit of the LIF neuron model is designed on FPAA Anadigm AD2 software (Fig. 1(c) of main paper). In AD2 software, the analog circuits can be implemented using a library of Configurable Analogue Modules (CAMs). The proposed LIF model in FPAA consists of four CAMs, sum/difference, integrator, comparator and differentiator CAMs.The specifications of each CAM are listed in Table S1.

2 BRAILLE AND MORSE CODE CHARACTER RECOGNISING SYSTEM

The proposed LIF-TMS neural network is implemented on a tactile sensing application for braille and morse code character identification system. 125 distinct braille characters and 62 morse codes are implemented with the proposed LIF-TMS crossbar array. The 187 characters are implemented using 6×2 LIF-TMS crossbar arrays. The LIF-TMS crossbar array's input layer acts as a tactile patch for the blind to press the braille and the morse code characters. In the Braille system, each character is represented by 6 dots (D1, D2, D3, D4, D5, D6) Chithra et al. (2022). In the case of Morse code, the repetitive combination of dots and dashes form alphabets and numbers. Here 10 dots are used for morse code, each column representing dots or dashes. the selection dots, D11 and D12, represent the braille and morse code selection. Each sensor-LIF block is represented by D_l. Here $l \in \{1, 2, 3...12\}$. Table 4 represents the characters implemented in the proposed tactile sensing system. The difference in braille and morse code character implementations is presented in Table S2.

3 RESULTS AND DISCUSSIONS

The proposed sensor-LIF module was designed in the SPICE tool with a circuit combination of summer, integrator, comparator and differentiator. Fig 4 shows the output of the designed Sensor-LIF circuit. The equivalent circuit of Flexi-force sensors as in Chithra et al. (2022) is used as the tactile sensor. The spike was generated for a reference voltage of $V_{ref} = 0.3V$, similar to the reference voltage obtained by stimulating the LIF neuron model with the touch sensor.

The equivalent circuit model of the sensor-LIF module is designed using Anadigm Designer 2 EDA (AD2) software. Fig. S2 shows the simulation results obtained from AD2 software for a pulse input of $100kHz$ frequency, amplitude $1V$ and time period 10 μ seconds. In Fig(S2), the Sum/diff CAM sums the analog input voltages, and the signal summation is from the input to the integrator CAM. The integrator CAM produces changing output voltage with a constant input voltage as presented in Fig S2. The comparator CAM compares the integrator output voltage and reference voltage from the tactile sensor. The comparator CAM output can have only high/low values, Fig S2. The differentiator CAM generates the instantaneous output of spikes as shown in Fig(S2).

The output of the LIF-TMS crossbar array is measured and used to train the subsequent dense layers for classification. A min-max scalar-based normalisation technique is adopted to remove the noise factors that might be affected at the boundary points. In min-max normalization, the noisy data is scaled up/down using a range based on averaging. The measured data is normalised into a symmetric range after preprocessing. For example, the data range of the measured value of character A is between 2.5 and 3.1. With min-max preprocessing, the data range for character A is limited between 2.7 and 2.9. This helps to remove the noise factors affecting the boundary points. The training for the sensor data is done with and without preprocessing technique.

The neural computations of each layer are computations done on myRIO FPGA. The FPGA processor is programmed using LABVIEW software. In the LABVIEW, we need to design real-time target VI to run on an ARM microprocessor and FPGA target VI to run on an FPGA processor. The Analog Input/Output (AIO) data read memory read, FIFO definition, and start and close of the FPGA VI target is done in the real-time target application. Whereas FPGA target application synthesis the circuit on FPGA and generates the bitstream file. The dense layer computations are done in the FPGA VI. The open FPGA VI reference will make the FPGA VI start running. The while loop makes the system continuously run for real-time applications. The single-cycle timed loop structures are always used in an FPGA VI, which will execute all functions within one tick of the clock; here, we use a 40 MHz global clock. The linear algebra matrix multiply function block and high throughput add function block work only inside the single-cycle timed loop. The output of a linear algebra matrix multiply function block is a column vector. Hence the bias values are converted to arrays for addition using high throughput add function block. The output of the presentation layer is the input to the subsequent layers. The trained ANN model contains 5 dense layers with input, hidden and output layers. The Relu activation function is used for all layers, and the softmax activation function is for the output layer. The testing accuracy for a system without the prepossessing method is 65% for braille and 75% for morse code. The performance accuracy is improved to 96% and 98% by using the preprocessed data.

REFERENCES

Chithra, R., Aswani, A. R., and James, A. P. (2022). Tms-crossbars with tactile sensing. *IEEE Transactions on Circuits and Systems II: Express Briefs* 69, 1842–1846

Dutta, S., Kumar, V., Shukla, A., Mohapatra, N. R., and Ganguly, U. (2017). Leaky integrate and fire neuron by charge-discharge dynamics in floating-body mosfet. *Scientific Reports* 7

4 SUPPLEMENTARY TABLES AND FIGURES

Table S1. Configurable Analogue Module (CAM) Specifications

CAM Name	Options	Parameters
Sum/Diff	Output Phase: Phase 1, Input 1: Non-inverting, Input 3: Off, Input 2: Inverting, Input 4: Off	Gain 1 (UpperInput): 1.00, Gain 2 (LowerInput): 1.00
Integrator	Polarity: Non-inverting Input, Sampling Phase: Phase 1, Compare Control, To: No Reset	Integration, Const.[1/us]: 0.05
Differentiator	Hold: On, Input, Phase: Phase 1	Differentiation, Const.[us]: 0.12
Comparator	Compare To: Dual Input, Input Sampling: Phase 1, Output Polarity: Inverted Hysteresis: 0 mV, Output Synch: Phase 1, Synch Clock Edge: Rising	

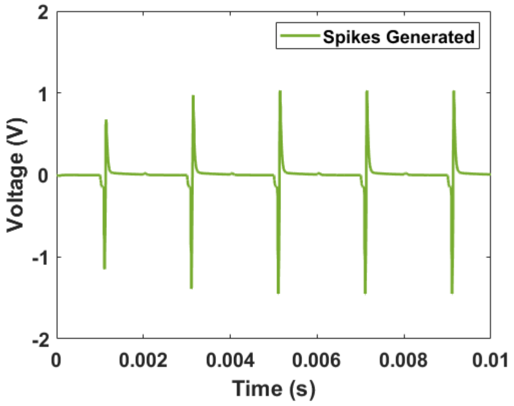

[]

[]

$\{D_{11}, D_{12}\}$	$\{D_7, D_8\}$	**Characters**
OFF,OFF	OFF,OFF	Braille Alphabet Capital (27 symbols)
OFF,OFF	OFF,ON	Braille Small (26 symbols)
OFF,OFF	ON,OFF	Braille Words (46 words)
OFF,OFF	ON,ON	Braille Numbers, punctuation & symbols (26 symbols)
ON,ON	Morse Alphabet Capital (26 symbols)	
OFF, ON	Morse Small (26 symbols)	
ON,ON	Morse code numbers (10 symbols)	

Figure S1. (a) Output of Sensor-LIF circuit: An ideal case (b) Braille and Morse code character implementation on LIF-TMS neural network

Table S2. Braille and Morse Character implementation on the sensor patch

Character	$\{D_1, D_2, D_3, D_4, D_5, D_6, D_7, D_8, D_9, D_{10}, D_{11}, D_{12}\}$
Braille Characters	
A	$\{ON, OFF, OFF, OFF, OFF, OFF, OFF, OFF, OFF, OFF, OFF, OFF\}$
a	$\{ON, OFF, OFF, OFF, OFF, OFF, ON, OFF, OFF, OFF, OFF, OFF\}$
1	$\{ON, OFF, OFF, OFF, OFF, OFF, ON, ON, OFF, OFF, OFF, OFF\}$
Morse Code	
A	$\{ON, OFF, OFF, ON, OFF, OFF, OFF, OFF, OFF, OFF, ON, ON\}$
a	$\{ON, OFF, OFF, ON, OFF, OFF, OFF, OFF, OFF, OFF, ON, OFF\}$
1	$\{ON, OFF, OFF, ON, OFF, ON, OFF, ON, OFF, ON, ON, ON\}$

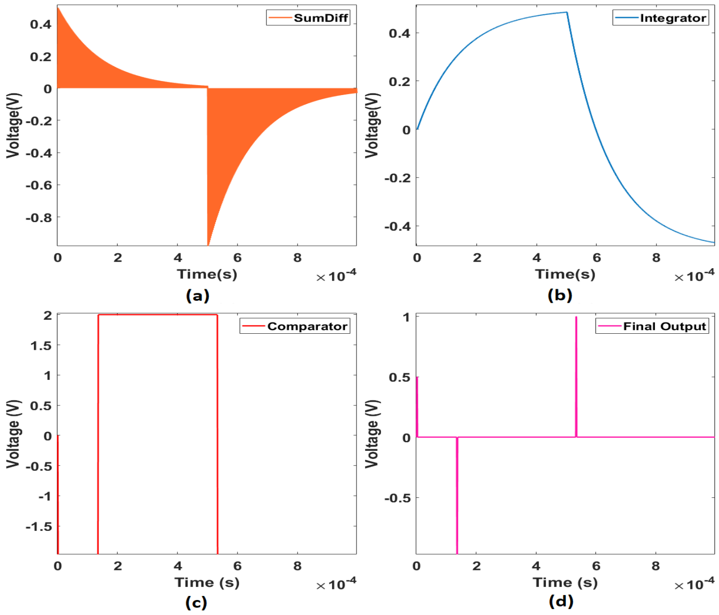

Figure S2. Output of Sensor-LIF circuit: FPAA Simulation using Anadigm Designer 2 EDA software (a) Sum/Diff CAM output (b) Integrator CAM output, (c) The Comparator CAM output with a reference voltage of 0.3V and (d) The Final output of the LIF
